# Elevated Neutrophil Percentage‐to‐Albumin Ratio Is Associated With Stroke‐Associated Pneumonia in Acute Ischemic Stroke: A Retrospective Cohort Study

**DOI:** 10.1002/brb3.71891

**Published:** 2026-11-05

**Authors:** Zheng Zhou, Jianmei Zhan, Haiyan Zheng, Lanzhen Wang, Tingting Duan, Ming Yang, Zhaoping Wu, Sijie Chen, Yueqi Xu, Jun Wang, Junnan Ye

**Affiliations:** ^1^ Department of Hematology The Quzhou Affiliated Hospital of Wenzhou Medical University, Quzhou People's Hospital Quzhou China; ^2^ Department of Neurology The Quzhou Affiliated Hospital of Wenzhou Medical University, Quzhou People's Hospital Quzhou China; ^3^ Department of Rehabilitation Medicine The Quzhou Affiliated Hospital of Wenzhou Medical University, Quzhou People's Hospital Quzhou China; ^4^ Department of Emergency The Quzhou Affiliated Hospital of Wenzhou Medical University, Quzhou People's Hospital Quzhou China

**Keywords:** acute ischemic stroke, inflammatory biomarkers, neutrophil percentage‐to‐albumin ratio, non‐linear modeling, risk stratification, stroke‐associated pneumonia

## Abstract

**Purpose:**

Stroke‐associated pneumonia (SAP) is a common and serious complication of acute ischemic stroke (AIS). The neutrophil percentage‐to‐albumin ratio (NPAR) is a composite inflammatory and nutritional marker derived from routine blood tests. This study investigated the association between NPAR and SAP and evaluated its functional form and incremental discriminatory value.

**Patients and Methods:**

This secondary analysis used a single‐center AIS registry that screened 2150 consecutive admissions between September 2016 and September 2022; 1766 patients comprised the NPAR analytic cohort. Associations between log_2_‐transformed NPAR and SAP were evaluated using multivariable logistic regression, generalized additive models (GAM), and two‐piecewise logistic regression.

**Results:**

SAP developed in 376 patients (21.3%). NPAR was independently associated with SAP after adjustment for the 18 prespecified covariates in Model 3 (adjusted odds ratio [OR] per doubling of NPAR = 6.14; 95% confidence interval [CI], 3.19–11.82; *p* < 0.001). Compared with the lowest quartile, the adjusted ORs for SAP were 1.16 (95% CI, 0.68–1.99), 1.72 (95% CI, 1.04–2.83), and 3.48 (95% CI, 2.08–5.83) across increasing NPAR quartiles (*p* for trend < 0.001). Generalized additive and piecewise models indicated an exploratory non‐linear, threshold‐associated pattern, with a data‐derived inflection point at approximately NPAR = 1.758. In ROC analysis, NPAR showed good discrimination (AUC = 0.785; 95% CI, 0.756–0.814), higher than the A2DS2 score alone (AUC = 0.758; *p* = 0.120) and lower than CRP alone (AUC = 0.826; *p* = 0.019). Adding NPAR to A2DS2 increased the apparent in‐sample AUC to 0.821 (*p* < 0.001), whereas adding NPAR to CRP increased it to 0.842 without a statistically significant increment over CRP alone (*p* = 0.213); these combination models were not internally validated.

**Conclusion:**

In this retrospective secondary analysis, NPAR was independently and non‐linearly associated with SAP after acute ischemic stroke. Multiple‐imputation analyses supported the robustness of these findings. NPAR provided complementary discriminatory information, particularly when added to A2DS2; however, independent external validation is required before clinical implementation.

## Graphical abstract

The image summarizes the study's core message: in 1,766 patients with acute ischemic stroke, the neutrophil percentage‐to‐albumin ratio (NPAR) was independently and non‐linearly associated with stroke‐associated pneumonia, with an exploratory inflection point at approximately NPAR = 1.758; NPAR also provided incremental discriminatory value when added to the A2DS2 score.

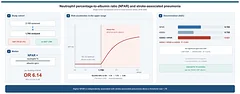

**Abbreviations**
A2DS2: Age, Atrial fibrillation, Dysphagia, Sex, Stroke severity
AF: atrial fibrillation
AIS: acute ischemic stroke
ALB: albumin
AUC: area under the curve
CI: confidence interval
COPD: chronic obstructive pulmonary disease
CRP: C‐reactive protein
eGFR: estimated glomerular filtration rate
FPG: fasting plasma glucose
GAM: generalized additive model
GCS: Glasgow Coma Scale
HbA1c: glycated hemoglobin
HCY: homocysteine
IQR: interquartile range
KWDT: Kubota Water Drinking Test
LDL‐C: low‐density lipoprotein cholesterol
MRI: magnetic resonance imaging
NIHSS: National Institutes of Health Stroke Scale
NLR: neutrophil‐to‐lymphocyte ratio
NPAR: neutrophil percentage‐to‐albumin ratio
NTC%: neutrophil percentage
OR: odds ratio
PLT: platelet count
ROC: receiver operating characteristic
SAP: stroke‐associated pneumonia
SD: standard deviation
SIDS: stroke‐induced immunodepression syndrome
SII: systemic immune‐inflammation index
TC: total cholesterol
TG: triglycerides
UA: uric acid
VIF: variance inflation factor
WBC: white blood cell count

## 1 Introduction

Globally, stroke ranks among the foremost causes of death and permanent disability, with AIS accounting for the majority of stroke cases (Banach et al. 2026; Golubnitschaja et al. 2024). Notwithstanding substantial progress in hyperacute stroke care, in‐hospital complications—most notably stroke‐associated pneumonia (SAP)—remain a major threat to patient recovery (Chaves et al. 2022). Occurring in roughly one‐in‐five to one‐in‐three AIS admissions, SAP prolongs hospitalization and substantially worsens functional prognosis and survival (de Jonge et al. 2022; Finlayson et al. 2011). Timely recognition of patients at elevated risk is accordingly indispensable for implementing targeted prophylactic measures and optimizing clinical outcomes.

A growing body of evidence highlights that post‐stroke immune dysregulation, compounded by nutritional compromise, substantially lowers the threshold for pulmonary infection by dismantling normal host‐defense homeostasis (Wang et al. 2023). Although widely deployed markers including CRP, WBC count, and the neutrophil‐to‐lymphocyte ratio (NLR) capture elements of systemic inflammation, none achieves adequate discriminatory accuracy for SAP in isolation (Wang et al. 2023; Xu et al. 2024). There is therefore a need for novel composite markers that integrate both inflammatory and nutritional dimensions of post‐stroke immune dysfunction.

The neutrophil percentage‐to‐albumin ratio (NPAR), calculated by dividing the neutrophil percentage (NTC%) by serum albumin concentration (g/L), is a readily available composite index derived entirely from standard blood tests (Gong et al. 2020). NPAR integrates two key pathophysiological axes: neutrophilia, reflecting innate immune activation and pro‐inflammatory responses (Thind et al. 2024; Wang et al. 2023); and hypoalbuminemia, reflecting nutritional depletion and impaired immune competence (Lyu et al. 2026). Both components are well‐established determinants of infection susceptibility and poor outcomes in critically ill patients. Beyond stroke, NPAR has been associated with Parkinson disease (Ke et al. 2025) and with pneumonia and poor outcome after spontaneous intracerebral hemorrhage (Lv et al. 2023). A recent study of 1008 patients with AIS reported that higher admission NPAR was linearly associated with SAP and that the association was stronger in men (Du et al. 2026).

The available evidence remains limited with respect to between‐cohort consistency in the functional form of the NPAR–SAP association. Du et al. reported no statistical evidence of non‐linearity, whereas the present study evaluated a possible threshold‐associated pattern using GAM and two‐piecewise logistic regression (Du et al. 2026). Further evaluation in an independent cohort is warranted because cohort characteristics, biomarker sampling time, SAP event frequency, model specification, and covariate adjustment may influence the observed exposure–response form. The incremental discrimination of NPAR beyond CRP and A2DS2 also remains incompletely characterized.

We therefore designed the present study around three pre‐specified objectives: (i) to determine whether early in‐hospital NPAR is independently associated with SAP after adjustment for established clinical and laboratory confounders; (ii) to characterize the functional form of the NPAR–SAP exposure–response relationship, specifically testing for a threshold‐associated pattern using GAM and two‐piecewise logistic regression; and (iii) to quantify the incremental discriminatory value of NPAR relative to CRP and A2DS2, both individually and in combination. The log_2_ transformation was chosen over log_10_ because it produces integer‐valued unit increments that correspond to biologically meaningful doublings of NPAR, facilitating interpretation of OR estimates.

## 2 Materials and Methods

### 2.1 Study Design and Participants

This single‐center retrospective study was a secondary analysis of a previously established acute ischemic stroke (AIS) registry at Quzhou People's Hospital, China. The registry screened 2150 consecutive admissions between September 2016 and September 2022. After application of the prespecified inclusion and exclusion criteria, 1766 patients comprised the present NPAR analytic cohort and were classified according to the occurrence of SAP within 7 days after stroke onset. No case–control matching was performed, and group differences were addressed using multivariable models. This study was approved by the Ethics Committee (Approval No. 2023–151), which waived informed consent because the study was retrospective and used de‐identified data.

The same institutional registry and ethics approval have supported related secondary analyses of SAP biomarkers, including the C‐reactive protein‐to‐albumin ratio (CAR), neutrophil‐to‐lymphocyte ratio (NLR), systemic immune‐inflammation index (SII), hemoglobin‐albumin‐lymphocyte‐platelet score (HALP), and platelet‐to‐lymphocyte ratio (PLR), as well as an analysis of CAR in relation to early neurological deterioration. These studies used overlapping or nested analytic subsets and should not be interpreted as independent validation cohorts. Published CAR‐SAP, NLR‐SAP, SII‐SAP, and HALP‐SAP reports are cited here (Duan et al. 2026; Duan et al. 2025; Wu et al. 2026; Zhang et al. 2026); the PLR‐SAP and CAR–early neurological deterioration analyses were under peer review at the time of this revision. The distinct exposure evaluated in the present study is NPAR, together with its non‐linear association with SAP and its comparative and incremental discrimination relative to CRP, SII, and A2DS2.

Inclusion criteria: (1) confirmed diagnosis of AIS within 24 h of symptom onset with admission during the same time window; (2) brain magnetic resonance imaging (MRI) performed within 48 h with radiological confirmation of acute infarction; (3) blood samples obtained within 48 h of symptom onset; and (4) complete clinical and laboratory data available for NPAR calculation.

Exclusion criteria: (1) incomplete medical records (*n*  =  112); (2) missing laboratory data required for NPAR calculation (*n*  =  69); (3) lost to follow‐up (*n*  =  35); yielding 1934 patients with complete data. These were further excluded if they had: (4) hemorrhagic transformation (*n*  =  45); (5) pre‐existing pneumonia at admission (*n*  =  68); (6) active malignancy (*n*  =  35); or (7) severe hepatic or renal dysfunction (*n*  =  20). The final analysis cohort comprised 1766 patients. The patient selection process is illustrated in Figure 1.

**FIGURE 1:**
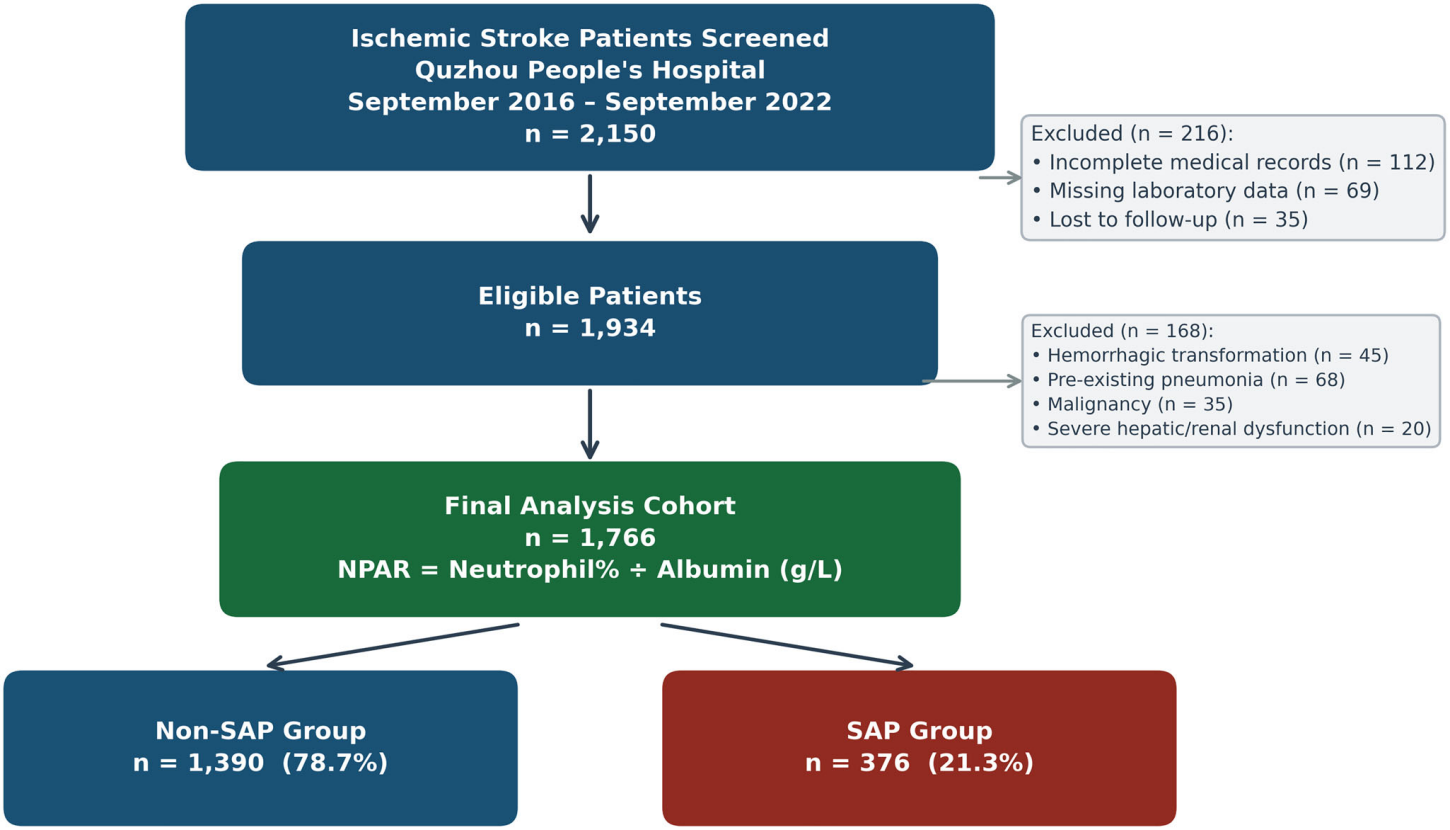
Study flowchart. A total of 2150 consecutively admitted ischemic stroke patients were screened; after applying exclusion criteria, 1766 patients were included in the final analysis.

### 2.2 Data Collection and Definitions

Baseline data were retrieved from medical records and included demographic and clinical characteristics at admission: age, sex, smoking status, hypertension, type 2 diabetes, atrial fibrillation (AF), and chronic obstructive pulmonary disease (COPD). Stroke severity was assessed using the National Institutes of Health Stroke Scale (NIHSS) (Cheng et al. 2023; Johansen et al. 2023; Larsen et al. 2022), swallowing difficulty was evaluated using the Kubota Water Drinking Test (KWDT) (Labeit et al. 2023; Labeit et al. 2024; Luo et al. 2024), and consciousness disturbances were assessed using the Glasgow Coma Scale (GCS) (Teasdale and Jennett 1974). We also recorded the need for nasal feeding tubes and the presence of consciousness disorders (Bodien et al. 2026). The A2DS2 score (Age, Atrial fibrillation, Dysphagia, Sex, Stroke Severity) was calculated as a validated composite predictor of SAP (Hoffmann et al. 2012).

Fasting venous blood samples were collected by trained nurses on the second morning after admission (approximately 6:00 AM) and processed within 2 h. Among all patients subsequently classified as having SAP, sampling preceded the first documented clinical evidence of pneumonia and antibiotic administration. Laboratory tests included WBC, neutrophil percentage (NTC%), absolute neutrophil count (NTC), lymphocyte count (LTC), platelet count (PLT), albumin (ALB), CRP, fasting plasma glucose (FPG), glycated hemoglobin (HbA1c), homocysteine (HCY), triglycerides (TG), total cholesterol (TC), low‐density lipoprotein cholesterol (LDL‐C), uric acid (UA), and serum creatinine. NPAR was calculated as NTC%/albumin (g/L) (Gong et al. 2020), and the systemic immune‐inflammation index (SII) was calculated as PLT × NTC/LTC. Albumin was expressed in g/L; most published NPAR studies, including the original report (Gong et al. 2020), express albumin in g/dL, so absolute NPAR values reported here are not directly comparable with those studies. The estimated glomerular filtration rate (eGFR) was calculated using the CKD‐EPI formula (Khandpur et al. 2022; Tarzi et al. 2025).

SAP diagnosis was determined independently by two attending neurologists following the revised criteria of the Centers for Disease Control and Prevention and the pneumonia recommendations of the Stroke Consensus Group (Smith et al. 2015). Only pneumonia first documented within 7 days after stroke onset was classified as SAP; pneumonia first documented later during hospitalization was not included in the analyzed outcome. Diagnostic criteria required at least one of: fever (>38°C) without alternative cause, abnormal WBC count, or altered mental status in patients ≥70 years; plus at least two respiratory criteria including purulent sputum, new cough or dyspnea, auscultatory findings, or oxygen desaturation. Radiologic confirmation required new or progressive infiltrates on chest CT.

### 2.3 Statistical Analysis

The normality of continuous variables was assessed using the Kolmogorov–Smirnov test. Normally distributed variables were expressed as mean ± standard deviation (SD) and compared with Student's *t*‐test; non‐normally distributed variables were presented as median (interquartile range, IQR) and compared using the Mann–Whitney *U* test. Categorical variables were analyzed using the chi‐square test.

NPAR values were log_2_‐transformed to reduce skewness and to permit interpretation per doubling. Because HbA1c had 287 missing values among the 1766 eligible patients, 20 imputed datasets (*m* = 20) were generated using stochastic regression imputation with age, sex, vascular risk factors, admission laboratory indices, stroke severity, thrombolysis, and SAP status as predictors. Thrombolysis was included only in the imputation model and was not a covariate in Model 3. Fully adjusted Model 3, subgroup models, and the segment‐specific coefficients from two‐piecewise logistic regression were estimated in each completed dataset and pooled using Rubin's rules. Descriptive and ROC analyses used the full cohort of 1766 patients. Model covariates were prespecified on clinical grounds and from reported SAP risk factors. VIFs were recalculated for the exact 18‐covariate Model 3 design matrix in each completed dataset; all mean VIFs were <5, with HbA1c having the highest mean VIF (3.070; range, 2.965–3.139; Table S1).

Three logistic regression models were constructed: Model 1, unadjusted; Model 2, adjusted for age and sex; and Model 3, fully adjusted for age, sex, smoking status, hypertension, diabetes mellitus, atrial fibrillation, COPD, fasting plasma glucose, HbA1c, triglycerides, total cholesterol, uric acid, homocysteine, CRP, albumin, eGFR, baseline NIHSS score, and KWDT results (18 covariates). Neither thrombolysis nor GCS was included in Model 3.

A generalized additive model (GAM) with a penalized cubic spline (five basis knots) was fitted in the first completed dataset to visualize the potentially non‐linear relationship between log_2_‐NPAR and SAP; a pointwise 95% bootstrap band was obtained from 200 resamples. For the two‐piecewise logistic analysis, a common inflection point was selected in the first completed dataset by maximizing the log‐likelihood within the fifth–90th percentile of log_2_‐NPAR and was then held fixed across all 20 completed datasets. Segment coefficients and standard errors were pooled using Rubin's rules. The difference between the two segment slopes was evaluated with a pooled 1‐degree‐of‐freedom Wald's test. Because the inflection point was selected from one completed dataset, no confidence interval is reported for the threshold and its interpretation is exploratory.

Predictive performance was evaluated using receiver operating characteristic (ROC) curves in the full cohort. AUC confidence intervals and pairwise AUC comparisons were calculated using the DeLong method. Sensitivity, specificity, and accuracy were calculated at the Youden‐index cutoff. The NPAR + CRP and NPAR + A2DS2 models were fitted and evaluated in the same cohort; their AUCs and incremental gains are apparent in‐sample estimates and were not included in the bootstrap validation. For each subgroup, a joint interaction model included log_2_‐NPAR, the subgroup indicator, their product term, and the Model 3 covariates except the stratifying variable. These models were fitted in all 20 completed datasets and pooled using Rubin's rules; interaction *p* values were obtained from pooled Wald's tests of the product‐term coefficient. No multiplicity adjustment was applied, so subgroup findings were treated as exploratory. Model 3 calibration used predictions averaged across the 20 completed datasets and was examined using a calibration plot and the Hosmer–Lemeshow goodness‐of‐fit test. Internal validation was confined to the fully adjusted Model 3 and used 200 bootstrap resamples in the first completed dataset to estimate optimism in the AUC.

## 3 Results

### 3.1 Baseline Characteristics by SAP Status

Among the 1766 AIS patients included in the final analysis (screened from 2150 initially identified patients), 376 (21.3%) developed SAP within 7 days after stroke onset (Table 1). Compared with the non‐SAP group, SAP patients were significantly older (74.49 ± 11.74 vs. 67.84 ± 12.26 years, *p* < 0.001), and more frequently had atrial fibrillation (35.1% vs. 11.5%) and COPD (15.7% vs. 4.0%, both *p* < 0.001). No significant differences were observed in sex distribution or smoking status.

**TABLE 1:** Baseline characteristics of patients with acute ischemic stroke, stratified by stroke‐associated pneumonia (SAP) status.

Variables	Overall (*N* = 1766)	Non‐SAP (*N* = 1390)	SAP (*N* = 376)	*p*‐value
*N*	1766	1390	376	
Age (years)	69.26 ± 12.45	67.84 ± 12.26	74.49 ± 11.74	**<0.001**
Temperature (°C)	36.50 ± 0.47	36.49 ± 0.37	36.54 ± 0.71	0.087
***Gender, n (%)***				
Male	1050 (59.5%)	839 (60.4%)	211 (56.1%)	0.153
Female	716 (40.5%)	551 (39.6%)	165 (43.9%)	
Smoke, *n* (%)	649 (36.7%)	511 (36.8%)	138 (36.7%)	1.000
Hypertension, *n* (%)	1357 (76.8%)	1070 (77.0%)	287 (76.3%)	0.845
Diabetes, *n* (%)	628 (35.6%)	497 (35.8%)	131 (34.8%)	0.789
Atrial fibrillation, *n* (%)	292 (16.5%)	160 (11.5%)	132 (35.1%)	**<0.001**
Nasal feeding, *n* (%)	177 (10.0%)	34 (2.4%)	143 (38.0%)	**<0.001**
Disturbance of consciousness, *n* (%)	124 (7.0%)	28 (2.0%)	96 (25.5%)	**<0.001**
COPD, *n* (%)	115 (6.5%)	56 (4.0%)	59 (15.7%)	**<0.001**
***Laboratory parameters***				
PLT (×10^9^/L)	189.00 (151.00–233.00)	188.00 (152.00–229.00)	190.00 (148.00–243.75)	0.304
NPAR	1.76 (1.54–2.01)	1.70 (1.50–1.91)	2.11 (1.85–2.37)	**<0.001**
FPG (mg/dL)	101.63 (90.10–128.44)	100.01 (89.38–124.83)	108.48 (93.12–138.89)	**<0.001**
HbA1C (%)	6.00 (5.50–7.20)	6.00 (5.50–7.20)	6.10 (5.60–7.30)	0.298
TG (mg/dL)	112.93 (79.93–155.00)	117.80 (84.14–163.85)	93.00 (69.08–127.76)	**<0.001**
TC (mmol/L)	4.28 (3.65–5.01)	4.31 (3.69–5.03)	4.18 (3.57–4.92)	**0.022**
LDL‐C (mmol/L)	2.78 (2.18–3.41)	2.80 (2.21–3.42)	2.73 (2.08–3.33)	0.129
UA (µmol/L)	311.95 (251.00–380.00)	313.00 (257.40–382.08)	303.75 (235.45–369.80)	**0.034**
HCY (µmol/L)	14.93 (11.71–19.20)	14.60 (11.50–19.00)	16.30 (12.93–20.02)	**<0.001**
CRP (mg/L)	2.82 (1.15–6.91)	2.00 (1.00–4.26)	13.62 (4.00–37.47)	**<0.001**
WBC (×10^9^/L)	7.49 ± 2.82	6.84 ± 2.09	9.88 ± 3.72	**<0.001**
NTC (×10^9^/L)	4.54 (3.49–6.18)	4.20 (3.30–5.32)	7.00 (5.15–9.12)	**<0.001**
Albumin (g/L)	37.95 ± 3.85	38.43 ± 3.57	36.21 ± 4.33	**<0.001**
eGFR (mL/min/1.73 m^2^)	99.30 (79.67–118.90)	100.66 (81.76–119.32)	93.86 (71.75–116.24)	**<0.001**
***Clinical characteristics***				
NIHSS	3.00 (1.00–6.00)	2.00 (1.00–5.00)	5.00 (2.00–13.00)	**<0.001**
KWDT	1.00 (1.00–1.00)	1.00 (1.00–1.00)	1.00 (1.00–4.00)	**<0.001**
A2DS2	3.00 (2.00–4.00)	2.00 (2.00–4.00)	4.00 (3.00–7.00)	**<0.001**
GCS	15.00 (14.00–15.00)	15.00 (15.00–15.00)	14.00 (12.00–15.00)	**<0.001**

*Note*: Data are presented as mean ± standard deviation for normally distributed continuous variables, median (interquartile range) for non‐normally distributed continuous variables, or *n* (%) for categorical variables. HbA1C statistics are based on available data (pairwise deletion).

*p*‐values were calculated using Student's *t*‐test or Mann–Whitney *U* test for continuous variables and chi‐square test for categorical variables. Bold *p*‐values indicate statistical significance (*p* < 0.05).

Abbreviations: A2DS2, Age, Atrial Fibrillation, Dysphagia, Sex, Stroke Severity; COPD, chronic obstructive pulmonary disease; CRP, C‐reactive protein; eGFR, estimated glomerular filtration rate; FPG, fasting plasma glucose; GCS, Glasgow Coma Scale; HbA1C, glycated hemoglobin; HCY, homocysteine; KWDT, Kubota Water Drinking Test; LDL‐C, low‐density lipoprotein cholesterol; NIHSS, National Institutes of Health Stroke Scale; NPAR, neutrophil percentage‐to‐albumin ratio; NTC, neutrophil count; PLT, platelet count; SAP, stroke‐associated pneumonia; TC, total cholesterol; TG, triglycerides; UA, uric acid; WBC, white blood cell count.

Laboratory findings revealed markedly elevated inflammatory markers in the SAP group. NPAR was significantly higher in SAP patients compared to non‐SAP patients (2.111 [1.848–2.373] vs. 1.702 [1.502–1.914], *p* < 0.001). CRP and WBC were also elevated, while albumin was lower in the SAP group (all *p* < 0.05). Additionally, fasting glucose and homocysteine levels were higher in the SAP group (all *p* < 0.05).

SAP patients exhibited more severe neurological impairment, as indicated by higher NIHSS scores (5.00 [2.00–13.00] vs. 2.00 [1.00–5.00], *p* < 0.001), higher A2DS2 scores (4.00 [3.00–7.00] vs. 2.00 [2.00–4.00], *p* < 0.001), and lower GCS scores (all *p* < 0.001).

### 3.2 Baseline Characteristics by NPAR Quartiles

Patient characteristics across NPAR quartiles are shown in Table 2. The incidence of SAP increased markedly across quartiles: 7.0% in Q1 (0.4607 ≤ NPAR ≤ 1.5382), 9.3% in Q2 (1.5382< NPAR ≤ 1.7648), 18.8% in Q3 (1.7648< NPAR ≤ 2.0107), and 50.0% in Q4 (2.0107< NPAR ≤ 4.6128; *p* < 0.001). Higher NPAR quartiles were associated with increasing prevalence of atrial fibrillation and COPD (both *p* < 0.001), as well as progressively higher CRP, fasting glucose, and WBC counts. Neurological scores, including NIHSS, A2DS2, and KWDT, increased with higher NPAR quartiles (all *p* < 0.001).

**TABLE 2:** Baseline characteristics of patients stratified by quartiles of neutrophil percentage‐to‐albumin ratio (NPAR).

Variables	Q1 (*N* = 442) 0.4607 ≤ NPAR ≤ 1.5382	Q2 (*N* = 441) 1.5382 < NPAR ≤ 1.7648	Q3 (*N* = 441) 1.7648 < NPAR ≤ 2.0107	Q4 (*N* = 442) 2.0107 < NPAR ≤ 4.6128	*p*‐value
*N*	442	441	441	442	
SAP, *n* (%)	31 (7.0%)	41 (9.3%)	83 (18.8%)	221 (50.0%)	**<0.001**
Age (years)	65.86 ± 12.81	67.84 ± 12.14	69.87 ± 11.66	73.47 ± 11.90	**<0.001**
Temperature (°C)	36.47 ± 0.38	36.51 ± 0.36	36.48 ± 0.61	36.56 ± 0.47	**0.032**
***Gender, n (%)***					
Male	256 (57.9%)	262 (59.4%)	265 (60.1%)	267 (60.4%)	0.879
Female	186 (42.1%)	179 (40.6%)	176 (39.9%)	175 (39.6%)	
Smoke, *n* (%)	181 (41.0%)	158 (35.8%)	149 (33.8%)	161 (36.4%)	0.158
Hypertension, *n* (%)	339 (76.7%)	349 (79.1%)	335 (76.0%)	334 (75.6%)	0.592
Diabetes, *n* (%)	179 (40.5%)	153 (34.7%)	149 (33.8%)	147 (33.3%)	0.091
Atrial fibrillation, *n* (%)	41 (9.3%)	50 (11.3%)	78 (17.7%)	123 (27.8%)	**<0.001**
Nasal feeding, *n* (%)	11 (2.5%)	10 (2.3%)	35 (7.9%)	121 (27.4%)	**<0.001**
Disturbance of consciousness, *n* (%)	10 (2.3%)	7 (1.6%)	27 (6.1%)	80 (18.1%)	**<0.001**
COPD, *n* (%)	11 (2.5%)	19 (4.3%)	32 (7.3%)	53 (12.0%)	**<0.001**
***Laboratory parameters***					
PLT (×10^9^/L)	192.00 (151.00–229.75)	195.00 (156.00–237.00)	190.00 (153.00–231.00)	180.50 (142.25–231.50)	0.072
NPAR	1.38 (1.27–1.47)	1.66 (1.60–1.71)	1.88 (1.83–1.95)	2.22 (2.10–2.38)	**<0.001**
FPG (mg/dL)	102.08 (91.18–126.82)	97.31 (88.48–122.36)	100.91 (90.10–127.94)	107.94 (91.90–136.86)	**<0.001**
HbA1C (%)	6.20 (5.60–7.45)	6.00 (5.50–7.00)	6.00 (5.60–7.10)	6.00 (5.50–7.30)	0.291
TG (mg/dL)	130.20 (92.11–177.80)	119.57 (89.46–162.08)	111.60 (81.48–153.23)	88.57 (67.31–124.88)	**<0.001**
TC (mmol/L)	4.49 (3.83–5.08)	4.31 (3.73–5.06)	4.26 (3.65–5.00)	4.12 (3.48–4.85)	**<0.001**
LDL‐C (mmol/L)	2.90 (2.27–3.50)	2.82 (2.22–3.44)	2.80 (2.23–3.40)	2.64 (2.03–3.24)	**0.002**
UA (µmol/L)	319.35 (267.23–384.80)	313.00 (257.80–384.50)	308.00 (250.00–374.10)	309.35 (235.25–375.80)	**0.043**
HCY (µmol/L)	14.16 (11.21–18.50)	14.70 (11.90–18.84)	14.75 (11.67–19.17)	16.30 (12.50–20.45)	**<0.001**
CRP (mg/L)	1.85 (1.00–3.26)	2.00 (1.00–4.20)	3.00 (1.19–6.81)	8.14 (3.00–22.69)	**<0.001**
WBC (×10^9^/L)	6.51 ± 2.20	6.81 ± 1.99	7.34 ± 2.26	9.29 ± 3.62	**<0.001**
NTC (×10^9^/L)	3.39 (2.70–4.15)	4.17 (3.43–5.08)	4.93 (3.94–6.25)	6.87 (5.22–9.07)	**<0.001**
Albumin (g/L)	40.11 ± 3.95	38.69 ± 2.65	37.90 ± 2.94	35.11 ± 3.85	**<0.001**
eGFR (mL/min/1.73 m^2^)	104.83 (84.13–122.86)	100.69 (82.12–116.73)	96.52 (77.22–118.68)	92.11 (75.37–116.82)	**<0.001**
***Clinical characteristics***					
NIHSS	2.00 (1.00–4.00)	2.00 (1.00–4.00)	3.00 (1.00–6.00)	4.00 (2.00–9.75)	**<0.001**
KWDT	1.00 (1.00–1.00)	1.00 (1.00–1.00)	1.00 (1.00–1.00)	1.00 (1.00–3.00)	**<0.001**
A2DS2	2.00 (2.00–3.00)	2.00 (2.00–4.00)	3.00 (2.00–4.00)	4.00 (3.00–6.00)	**<0.001**
GCS	15.00 (15.00–15.00)	15.00 (15.00–15.00)	15.00 (14.00–15.00)	15.00 (12.00–15.00)	**<0.001**

*Note*: Values are presented as mean ± standard deviation for normally distributed continuous variables, median (interquartile range) for non‐normally distributed continuous variables, or *n* (%) for categorical variables. HbA1C statistics are based on available data (pairwise deletion).

*p*‐values for continuous variables were calculated using one‐way ANOVA or Kruskal–Wallis test; for categorical variables, chi‐square test was used. Bold *p*‐values indicate statistical significance (*p* < 0.05).

Abbreviations: A2DS2, Age, Atrial Fibrillation, Dysphagia, Sex, Stroke Severity; COPD, chronic obstructive pulmonary disease; CRP, C‐reactive protein; eGFR, estimated glomerular filtration rate; FPG, fasting plasma glucose; GCS, Glasgow Coma Scale; HbA1C, glycated hemoglobin; HCY, homocysteine; KWDT, Kubota Water Drinking Test; LDL‐C, low‐density lipoprotein cholesterol; NIHSS, National Institutes of Health Stroke Scale; NPAR, neutrophil percentage‐to‐albumin ratio; NTC, neutrophil count; PLT, platelet count; SAP, stroke‐associated pneumonia; TC, total cholesterol; TG, triglycerides; UA, uric acid; WBC, white blood cell count.

### 3.3 Non‐Linear Association and Threshold Effect of NPAR on SAP Risk

A penalized cubic‐spline GAM visualization supported a curved association between log_2_‐NPAR and SAP risk: the fitted probability was relatively flat at lower NPAR values and increased more steeply above the data‐derived inflection point of approximately 1.758 (Figure 4). Because the curve and cut‐point were derived from the first completed dataset, this pattern should be considered exploratory.

Using the common data‐derived inflection point (NPAR = 1.758), the pooled two‐piecewise model showed no statistically significant association below the inflection point (adjusted OR = 0.61; 95% CI, 0.26–1.43; *p* = 0.259) and a strong association above it (adjusted OR = 97.42; 95% CI, 32.07–295.91; *p* < 0.001). The difference between the two segment slopes was supported by a pooled Wald's test (*χ* ^2^ = 39.13, 1 df; *p* < 0.001; Tables 3 and S2).

**TABLE 3:** Threshold‐associated and linear associations between log_2_‐transformed NPAR and stroke‐associated pneumonia after multiple imputation.

SAP outcome	Adjusted OR (95% CI)	*p*‐value
Model 1: Linear association (log_2_‐NPAR, per 1‐unit increase)		
NPAR (Model 1)	6.14 (3.19–11.82)	**<0.001**
Model 2: Two‐piecewise logistic regression		
Inflection point (*K*) log_2_‐NPAR = 0.814 (NPAR = 1.758)	—	—
NPAR < 1.758 (below K)	0.61 (0.26–1.43)	0.259
NPAR ≥ 1.758 (above K)	97.42 (32.07–295.91)	**<0.001**
Pooled Wald's test for difference in segment slopes	*χ* ^2^ = 39.13	**<0.001**

*Note*: Multivariable logistic regression models evaluating linear and two‐piecewise associations between log_2_‐transformed NPAR and SAP in the multiply imputed analytic cohort (*N* = 1766; SAP = 376).

Model 1 assumes a linear effect. Model 2 uses a common data‐derived inflection point at log_2_‐NPAR = 0.814 (NPAR = 1.758). This point was selected by maximum log‐likelihood within the 5th–90th percentile search range in the first completed dataset and was then held fixed across all 20 completed datasets; no confidence interval is reported for this exploratory threshold.

Both models were adjusted for age, gender, smoking, hypertension, diabetes, atrial fibrillation, COPD, FPG, HbA1c, TG, TC, UA, HCY, CRP, albumin, eGFR, NIHSS, and KWDT. NTC, WBC, NTC%, LDL‐C, and A2DS2 were excluded on the basis of mathematical overlap, collinearity, or model interpretability.

Segment ORs represent the association per 1‐unit increase in log_2_‐NPAR within each segment. Segment coefficients and standard errors were pooled across the 20 completed datasets using Rubin's rules.

The pooled Wald's test evaluates the difference between the two segment slopes (*χ* ^2^, 1 df).

Bold values indicate statistical significance (*p* < 0.05). —, not applicable.

Abbreviations: CI, confidence interval; *K*, inflection point; NPAR, neutrophil percentage‐to‐albumin ratio; NTC%, neutrophil percentage; OR, odds ratio; SAP, stroke‐associated pneumonia.

### 3.4 Association Between Log_2_‐NPAR and SAP: Multivariable Logistic Regression

To quantify the adjusted linear association of NPAR, multivariable logistic regression was performed with log_2_‐transformed NPAR. In the fully adjusted model based on multiply imputed data (*N* = 1766), each doubling of NPAR was associated with 6.14‐fold higher odds of SAP (95% CI, 3.19–11.82; *p* < 0.001) after adjustment for the 18 Model 3 covariates listed in Section 2 and Table 4; thrombolysis and GCS were not included (Table 4).

**TABLE 4:** Multivariable logistic regression analysis of the association between log2‐transformed NPAR and stroke‐associated pneumonia (SAP) after multiple imputation for missing HbA1c.

Exposure	Crude model (Model 1) OR (95% CI)	*p*‐value	Partially adjusted model (Model 2) OR (95% CI)	*p*‐value	Fully adjusted model (Model 3) OR (95% CI)	*p*‐value
**log2‐NPAR (per 1‐unit increase)**						
NPAR (continuous) *N* = 1766	52.45 (30.94–88.93)	**<0.001**	41.24 (24.08–70.63)	**<0.001**	6.14 (3.19–11.82)	**<0.001**
**NPAR quartile**						
Q1 (0.4607 ≤ NPAR ≤ 1.5382) *N* = 442, SAP = 31 (7.0%)	1.00 (Reference)		1.00 (Reference)		1.00 (Reference)	
Q2 (1.5382 < NPAR ≤ 1.7648) *N* = 441, SAP = 41 (9.3%)	1.36 (0.84–2.21)	0.2165	1.29 (0.79–2.10)	0.3138	1.16 (0.68–1.99)	0.5829
Q3 (1.7648 < NPAR ≤ 2.0107) *N* = 441, SAP = 83 (18.8%)	3.07 (1.99–4.75)	**<0.001**	2.77 (1.78–4.30)	**<0.001**	1.72 (1.04–2.83)	**0.0352**
Q4 (2.0107 < NPAR ≤ 4.6128) *N* = 442, SAP = 221 (50.0%)	13.26 (8.80–19.98)	**<0.001**	11.07 (7.30–16.79)	**<0.001**	3.48 (2.08–5.83)	**<0.001**
*p* for trend		**<0.001**		**<0.001**		**<0.001**

*Note*: Model 1: Unadjusted (crude model). Model 2: Adjusted for age and gender. Model 3: Adjusted for age, gender, smoking status, hypertension, diabetes mellitus, atrial fibrillation, COPD, fasting plasma glucose, HbA1c, triglycerides, total cholesterol, uric acid, homocysteine, C‐reactive protein, albumin, eGFR, baseline NIHSS score, and KWDT (18 covariates); thrombolysis and GCS were not included.

HbA1c missing values were handled using multiple imputation (*m* = 20). Model 3 estimates were fitted separately in each imputed dataset and pooled using Rubin's rules. The analytic cohort included 1766 patients. *p* for trend was calculated by entering the quartile number (1–4) as a continuous ordinal variable in the logistic regression model. Bold ORs and *p*‐values indicate statistical significance (*p* < 0.05).

Abbreviations: COPD, chronic obstructive pulmonary disease; CRP, C‐reactive protein; eGFR, estimated glomerular filtration rate; FPG, fasting plasma glucose; HbA1c, glycated hemoglobin; HCY, homocysteine; KWDT, Kubota Water Drinking Test; NIHSS, National Institutes of Health Stroke Scale; TC, total cholesterol; TG, triglycerides; UA, uric acid; OR, odds ratio; CI, confidence interval; NPAR, neutrophil percentage‐to‐albumin ratio; SAP, stroke‐associated pneumonia.

Quartile‐based analysis corroborated this dose–response relationship. Compared with patients in the lowest NPAR quartile, those in the second, third, and fourth quartiles had adjusted ORs of 1.16 (95% CI, 0.68–1.99; *p* = 0.583), 1.72 (95% CI, 1.04–2.83; *p* = 0.035), and 3.48 (95% CI, 2.08–5.83; *p* < 0.001), respectively (*p* for trend < 0.001; Table 4).

### 3.5 Subgroup Analyses

Exploratory subgroup models were fitted in each of the 20 completed datasets and pooled using Rubin's rules (Figure 2). The positive NPAR–SAP association was statistically significant in 11 of 14 strata. Nominal interactions were observed for age (OR = 14.99 [95% CI, 4.39–51.18] for <70 years and 3.82 [1.76–8.30] for ≥70 years; *p* for interaction = 0.039), sex (OR = 8.93 [3.47–22.99] in men and 3.54 [1.43–8.74] in women; *p* for interaction = 0.010), and atrial fibrillation (OR = 7.37 [3.34–16.27] without atrial fibrillation and 2.89 [0.81–10.33] with atrial fibrillation; *p* for interaction = 0.044). Interactions for smoking, hypertension, diabetes, and COPD were not statistically significant. Because no multiplicity adjustment was applied and several strata had wide confidence intervals, these findings are hypothesis‐generating.

**FIGURE 2:**
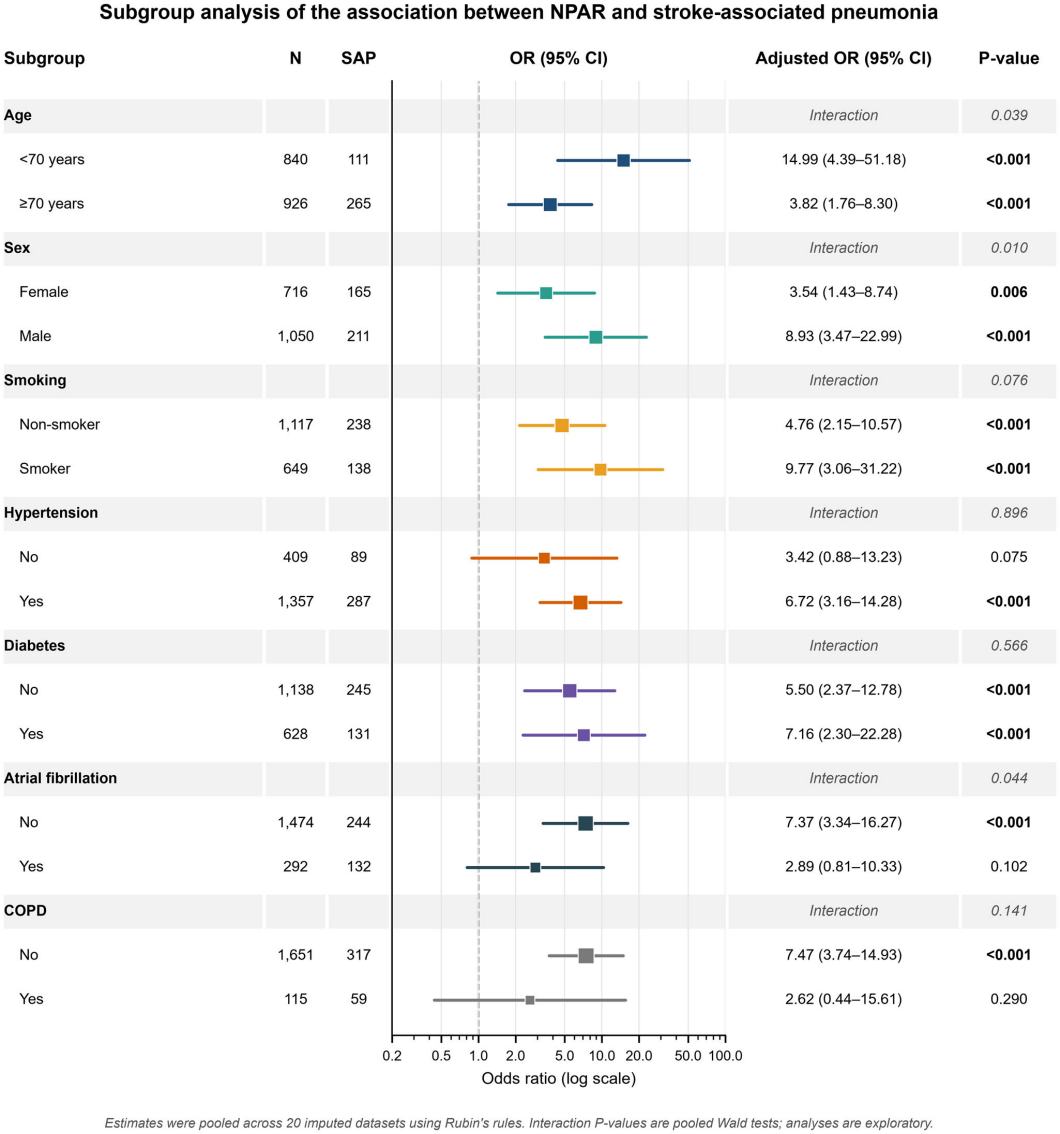
Multiple‐imputation subgroup analysis of the association between NPAR and stroke‐associated pneumonia (SAP). Adjusted odds ratios (ORs) represent a 1‐unit increase in log_2_‐NPAR (equivalent to a doubling of NPAR) within each prespecified stratum. For each subgroup, the joint model included log_2_‐NPAR, the subgroup indicator, their product term, and the Model 3 covariates except the stratifying variable. Models were fitted in all 20 completed datasets and pooled using Rubin's rules; interaction *p* values are pooled Wald's tests of the product‐term coefficient and therefore are not calculated from the displayed stratum‐specific ORs. *N* and SAP counts refer to the full cohort (*N* = 1766; SAP = 376). No multiplicity adjustment was applied; all subgroup findings are exploratory.

### 3.6 Predictive Performance of NPAR, CRP, and A2DS2 for SAP

Having established the independent association between NPAR and SAP, we next evaluated whether NPAR provided useful discriminatory information. In ROC analysis, NPAR alone achieved an AUC of 0.785 (95% CI, 0.756–0.814), with a sensitivity of 68.6% and specificity of 77.2% (Table 5 and Figure 3). CRP alone showed a higher AUC than NPAR (0.826; 95% CI, 0.800–0.851; *p* = 0.019), whereas the A2DS2 score alone showed a similar AUC (0.758; 95% CI, 0.729–0.787; *p* = 0.120). Adding NPAR to CRP increased the apparent in‐sample AUC to 0.842 (95% CI, 0.816–0.868), and adding NPAR to A2DS2 increased it to 0.821 (95% CI, 0.794–0.847). The increase over A2DS2 alone was statistically significant (*p* < 0.001), whereas the increase over CRP alone was not (*p* = 0.213). These combination‐model AUCs and incremental gains were not internally validated; bootstrap internal validation was performed only for the fully adjusted Model 3. The Hosmer–Lemeshow test did not detect significant lack of fit in the derivation cohort (*χ* ^2^ = 9.37; *p* = 0.312; Figure 4b and Table S2).

**TABLE 5:** Apparent in‐sample discrimination of NPAR, CRP, A2DS2, and combination models for stroke‐associated pneumonia.

Model	AUC	95% CI (AUC)	Sensitivity (%)	Specificity (%)	Accuracy (%)	DeLong *p*‐value
NPAR	0.785	0.756–0.814	68.6	77.2	75.4	Reference
CRP	0.826	0.800–0.851	66.8	86.4	82.2	vs. NPAR: *p* = 0.019
A2DS2	0.758	0.729–0.787	48.1	90.5	81.5	vs. NPAR: *p* = 0.120
NPAR + CRP	0.842	0.816–0.868	69.1	89.8	85.4	vs. CRP: *p* = 0.213; vs. NPAR: *p* < 0.001
NPAR + A2DS2	0.821	0.794–0.847	68.9	83.7	80.5	vs. A2DS2: *p* < 0.001

*Note*: This table summarizes apparent in‐sample diagnostic performance of log_2_‐transformed NPAR, CRP, A2DS2, and their combinations for SAP in the full acute ischemic stroke cohort (*N* = 1766; SAP = 376).

The ROC predictors in this table do not directly include HbA1c; therefore, results were identical across the 20 imputed datasets. The imputed workbook was used to retain the full analytic cohort.

The combined models (NPAR + CRP and NPAR + A2DS2) were fitted and evaluated in the same cohort using logistic‐regression predicted probabilities as the ROC score. Their AUCs and incremental gains are apparent in‐sample estimates and were not internally validated; bootstrap validation was confined to the fully adjusted Model 3 reported in Table S2 and Figure 4c.

Sensitivity and specificity were determined at the optimal cut‐off point using the Youden index. AUC 95% CIs and statistical comparisons between AUCs were estimated using the DeLong method.

Abbreviations: A2DS2, Age, Atrial Fibrillation, Dysphagia, Stroke Severity and Sex; AUC, area under the ROC curve; CI, confidence interval; CRP, C‐reactive protein; NPAR, neutrophil percentage‐to‐albumin ratio; ROC, receiver operating characteristic; SAP, stroke‐associated pneumonia.

**FIGURE 3:**
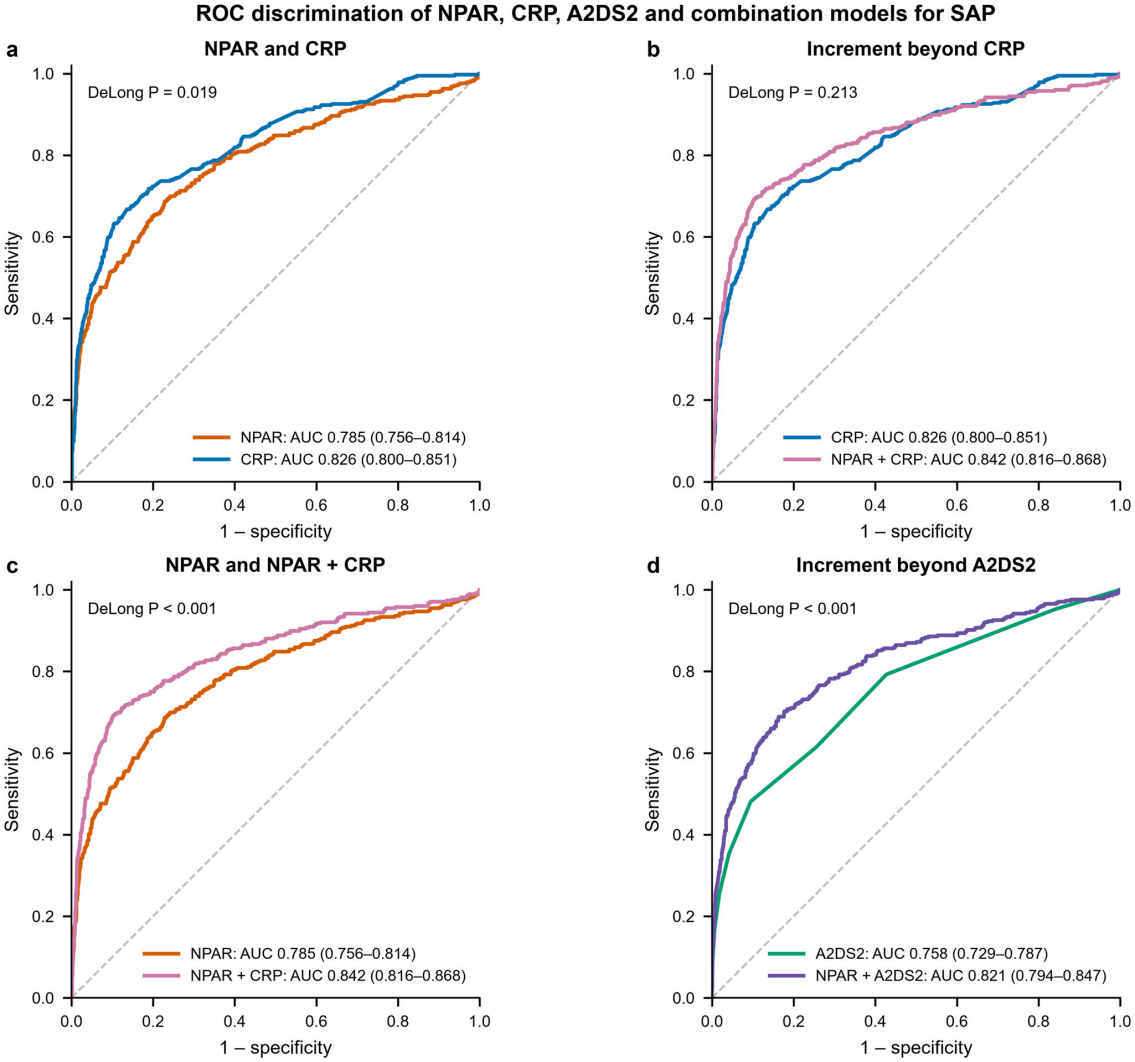
Full‐cohort ROC comparisons for stroke‐associated pneumonia (*N* = 1766; SAP = 376). (a) NPAR versus CRP (AUC 0.785 vs. 0.826; DeLong *p* = 0.019). (b) CRP versus NPAR + CRP (AUC 0.826 vs. 0.842; *p* = 0.213). (c) NPAR versus NPAR + CRP (AUC 0.785 vs. 0.842; *p* < 0.001). (d) A2DS2 versus NPAR + A2DS2 (AUC 0.758 vs. 0.821; *p* < 0.001). Parentheses in the figure give 95% confidence intervals. Pairwise comparisons used DeLong's method. The combination‐model AUCs and incremental gains are apparent in‐sample estimates and were not internally validated; bootstrap internal validation was confined to the fully adjusted Model 3 shown in Figure 4c.

**FIGURE 4:**
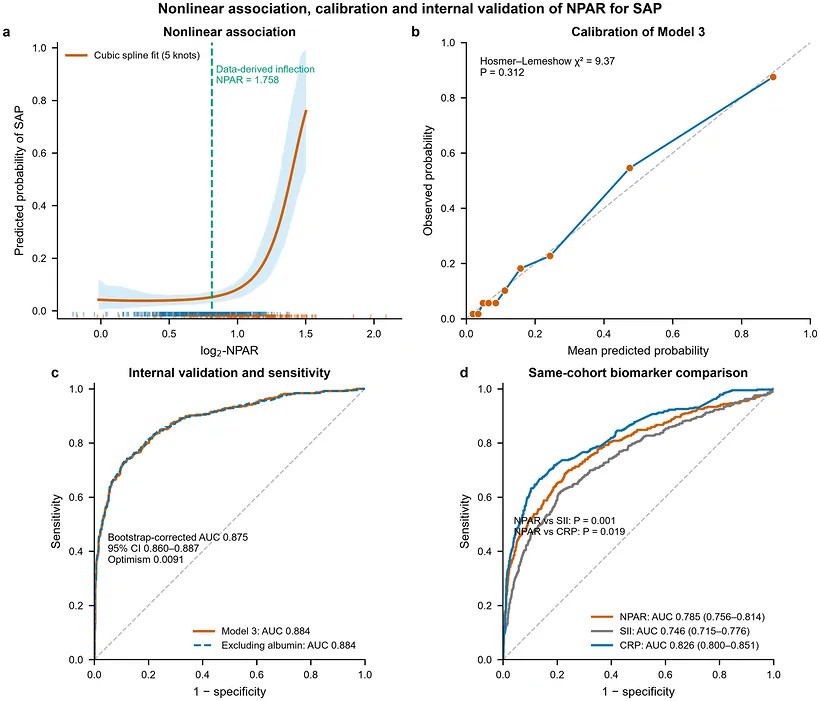
Nonlinear association, calibration, internal validation, and same‐cohort biomarker comparison for stroke‐associated pneumonia. (a) Penalized cubic‐spline GAM fitted in the first completed dataset, with a pointwise 95% band from 200 bootstrap resamples and the exploratory data‐derived inflection point at NPAR = 1.758. (b) Calibration plot for Model 3 using predictions averaged across the 20 completed datasets. The Hosmer–Lemeshow test did not detect significant lack of fit in the derivation cohort (*χ* ^2^ = 9.37; *p* = 0.312). (c) Model 3 discrimination and albumin‐exclusion sensitivity analysis; the apparent AUC was 0.884 and the bootstrap optimism‐corrected AUC was 0.875 (95% CI, 0.860–0.887; 200 resamples in the first completed dataset). (d) Same‐cohort ROC comparison of NPAR, SII, and CRP (AUCs 0.785, 0.746, and 0.826, respectively; NPAR vs. SII *p* = 0.001; NPAR vs. CRP *p* = 0.019). The inflection point is exploratory and is not a validated clinical cut‐off.

## 4 Discussion

Our principal finding is that NPAR, an integrated index of neutrophil burden and nutritional status, was independently and non‐linearly associated with SAP in patients with acute ischemic stroke. After multiple imputation of missing HbA1c values, each doubling of NPAR was associated with a 6.14‐fold increase in the odds of SAP, and the highest NPAR quartile had 3.48‐fold higher odds than the lowest quartile. The consistency of the association across linear, quartile‐based, and piecewise models supports NPAR as an informative risk marker within this cohort.

The biological rationale for NPAR may reflect complementary information carried by its two components. Neutrophil percentage may rise after AIS as part of sterile inflammatory and neuroendocrine responses, and excessive neutrophil activation could contribute to pulmonary vulnerability through oxidative and extracellular‐trap pathways (Vallés et al. 2017; Wang et al. 2023). Lower albumin may reflect reduced nutritional reserve and antioxidant capacity and could be associated with impaired host defense (Lv et al. 2023; Thind et al. 2024). These pathways were not measured directly and therefore remain plausible explanations rather than demonstrated mechanisms. Because NPAR itself was not included in the VIF analysis, the albumin VIF cannot establish weak correlation or biological independence between the components. Instead, the similar estimate after removing albumin from the adjustment set (OR = 6.97; 95% CI, 3.94–12.33) suggests that the association was not solely attributable to simultaneous adjustment for albumin.

The NPAR–SAP association appeared non‐linear. Risk increased most steeply above the data‐derived inflection point of approximately 1.758. In the piecewise model, the association below this value was not statistically significant, whereas the association above it was strong (adjusted OR = 97.42; 95% CI, 32.07–295.91; *p* < 0.001). The very large upper‐segment estimate and wide confidence interval may reflect numerical or model instability and should not be interpreted as a stable clinical effect size. Similar results were observed when albumin was removed from the adjustment set, reducing concern that the finding was solely a mathematical consequence of including albumin both in NPAR and as a covariate. Because the inflection point was estimated in a single‐center retrospective cohort, it should be interpreted as an exploratory threshold‐associated pattern rather than a clinically validated decision cutoff.

A recently published AIS cohort study by Du et al. (2026) also found a positive NPAR–SAP association but reported no statistical evidence of non‐linearity (*p* for nonlinearity = 0.80). That study further identified sex‐related effect modification, with a stronger association in men. Our exploratory subgroup analysis likewise yielded a nominal sex interaction (*p* for interaction = 0.010), with a stronger association in men, whereas our functional‐form analysis suggested a threshold‐associated pattern. Differences in cohort characteristics, NPAR sampling time, SAP event frequency, modeling strategy, and covariate adjustment may contribute to the differing functional forms, but these explanations cannot be established from the available data. Neither study provides independent validation of the other.

Head‐to‐head ROC comparison suggested that NPAR has complementary discriminatory value within this cohort. NPAR alone showed good discrimination (AUC = 0.785), lower than CRP alone but comparable with A2DS2. Adding NPAR to A2DS2 increased the apparent in‐sample AUC from 0.758 to 0.821, whereas the increase after adding NPAR to CRP was numerically modest and not statistically significant. Because these combination models were developed and evaluated in the same cohort and were not internally validated, the gains may be optimistic. NPAR may therefore be most useful for hypothesis generation alongside clinical risk scores rather than as a replacement for established inflammatory markers.

The exploratory subgroup analyses yielded nominal interactions with age, sex, and atrial fibrillation. The NPAR–SAP association appeared stronger among patients aged <70 years, men, and patients without atrial fibrillation. These patterns may reflect clinical or immunological differences, but they were identified across multiple comparisons, several stratum‐specific estimates were imprecise, and none has been externally validated. They should therefore be interpreted as hypothesis‐generating rather than as evidence for subgroup‐specific clinical thresholds.

To contextualize NPAR against another inflammation‐related composite marker evaluated in the same registry, we compared it with SII. NPAR showed a higher AUC than SII (0.785 vs. 0.746; *p* = 0.001). This within‐cohort comparison supports the relevance of albumin‐adjusted neutrophil burden for SAP discrimination but does not represent external validation.

The present findings should also be interpreted in relation to other biomarker analyses derived from the same institutional AIS registry. Prior reports evaluated CAR, NLR, SII, and HALP in relation to SAP (Duan et al. 2026; Duan et al. 2025; Wu et al. 2026; Zhang et al. 2026), while PLR‐SAP and CAR–early neurological deterioration analyses were under review at the time of this revision. These reports represent related secondary analyses with overlapping or nested patient subsets rather than independent replications. The specific contribution of the current study is the evaluation of NPAR, which integrates neutrophil percentage with albumin, using multiple imputation, non‐linear and piecewise modeling, direct comparison with SII, CRP, and A2DS2, and assessment of incremental discrimination. Accordingly, the findings establish a biomarker‐specific association within a previously reported registry and require validation in an independent external cohort.

Several limitations should be acknowledged. First, this was a single‐center retrospective study. Important SAP determinants, including infarct location and volume, aspiration events, mechanical ventilation, stroke subtype, antibiotic exposure, oral hygiene, pre‐stroke functional status, and frailty, were unavailable or were not included; residual confounding by these factors therefore remains possible. The same institutional registry has also supported other biomarker analyses with overlapping or nested cohorts; consequently, those studies are related secondary analyses rather than independent replications, and their findings may be correlated. Second, SAP diagnosis was based on clinical and radiographic documentation, and microbiological confirmation was not available for all cases. Third, HbA1c had 287 missing values; multiple imputation was used to retain the full cohort in fully adjusted analyses, but this approach assumes that missingness can be explained by observed variables. Fourth, NPAR was measured only once, on the second morning after admission, and dynamic changes during hospitalization were not assessed. Although samples in all patients who developed SAP were obtained before the first documented clinical evidence of pneumonia and before antibiotic administration, this temporal ordering does not exclude the presence of an evolving subclinical infection at sampling. Therefore, part of the observed association may reflect early infection‐related changes rather than only antecedent susceptibility. Fifth, the NPAR + CRP and NPAR + A2DS2 models were developed and evaluated in the same cohort; their AUCs and incremental gains are apparent in‐sample estimates, and only the fully adjusted Model 3 underwent bootstrap internal validation. Finally, independent prospective multicenter validation is required before NPAR can be incorporated into routine SAP risk prediction.

## 5 Conclusion

In this retrospective secondary analysis of 1766 patients with acute ischemic stroke, NPAR was independently and non‐linearly associated with SAP. Multiple‐imputation analyses supported the robustness of the association after accounting for missing HbA1c values. NPAR provided complementary discriminatory information, with a statistically significant incremental value when added to A2DS2; the incremental improvement beyond CRP alone was not statistically significant. Independent external validation is required before clinical implementation.

## Author Contributions

ZZ and JZ conceptualized and designed the study. LW, SC, and HZ collected the data. TD and ZW performed data analysis and interpretation. ZZ drafted the initial manuscript. MY, LW, and YX prepared the figures and tables. JY critically revised the manuscript for important intellectual content. All authors reviewed and approved the final version and are accountable for all aspects of the work.

## Funding

The authors have nothing to report.

## Ethics Statements

This secondary analysis of a previously established institutional AIS registry adhered to the principles of the Declaration of Helsinki. The protocol was approved by the Ethics Committee of Quzhou Affiliated Hospital, Wenzhou Medical University, China (Approval No. 2023–151). Because the study was retrospective and contained no identifiable data, informed consent was waived. Patients’ privacy and personal identity information were protected.

## Conflicts of Interest

The authors have no conflicts of interest in this work.

## Supporting information




**Table S1**: Variance inflation factors for the exact final Model 3 covariates after multiple imputation.

**Table S2**: Model robustness and sensitivity analyses based on multiply imputed data. Analyses included albumin‐adjustment sensitivity, piecewise threshold modeling, bootstrap internal validation, ROC comparisons with SII and CRP, and calibration assessment.

## Data Availability

The datasets used and analyzed during this study are available from the corresponding author on reasonable request.
