# Supplementary material for: Elevated Neutrophil Percentage‐to‐Albumin Ratio Is Associated With Stroke‐Associated Pneumonia in Acute Ischemic Stroke: A Retrospective Cohort Study
Source: Brain Behav. 2026 Nov 5;16(11):e71891. doi: 10.1002/brb3.71891 (PMC13740347; doi:10.1002/brb3.71891)
Supplement: Supplementary file 1 [file BRB3-16-e71891-s001.docx]

**Supplementary Table S1. Variance inflation factors for the exact final Model 3 covariates after multiple imputation**

| **No.** | **Final Model 3 covariate** | **VIF, mean (range)** | **Interpretation** |
| --- | --- | --- | --- |
| 1 | Age (years) | 1.414 (1.413–1.415) | No collinearity |
| 2 | Gender | 1.782 (1.780–1.785) | No collinearity |
| 3 | Smoking status | 1.728 (1.723–1.731) | No collinearity |
| 4 | Hypertension | 1.066 (1.065–1.068) | No collinearity |
| 5 | Diabetes mellitus | 1.884 (1.849–1.918) | No collinearity |
| 6 | Atrial fibrillation | 1.176 (1.176–1.177) | No collinearity |
| 7 | COPD | 1.120 (1.119–1.121) | No collinearity |
| 8 | FPG (mg/dL) | 2.435 (2.381–2.461) | Mild collinearity |
| 9 | HbA1c (%) | 3.070 (2.965–3.139) | Mild collinearity |
| 10 | TG (mg/dL) | 1.235 (1.234–1.236) | No collinearity |
| 11 | TC (mmol/L) | 1.205 (1.202–1.210) | No collinearity |
| 12 | UA (µmol/L) | 1.346 (1.343–1.353) | No collinearity |
| 13 | HCY (µmol/L) | 1.106 (1.105–1.107) | No collinearity |
| 14 | CRP (mg/L) | 1.144 (1.144–1.146) | No collinearity |
| 15 | Albumin (g/L) | 1.154 (1.151–1.159) | No collinearity |
| 16 | eGFR (mL/min/1.73 m²) | 1.440 (1.439–1.443) | No collinearity |
| 17 | NIHSS | 1.648 (1.641–1.655) | No collinearity |
| 18 | KWDT | 1.736 (1.732–1.741) | No collinearity |

*VIF values were recalculated using the exact 18-covariate final Model 3 design matrix in each of the 20 completed datasets (N = 1,766 per dataset) and are reported as mean (range). SAP (outcome) and NPAR (primary exposure) were not included in the VIF calculation.*

*VIF < 2 indicates no collinearity; 2 ≤ VIF < 5 indicates mild collinearity. All mean VIFs were <5. HbA1c had the highest mean VIF: 3.070 (range, 2.965–3.139).*

*The final Model 3 covariates were age, gender, smoking status, hypertension, diabetes mellitus, atrial fibrillation, COPD, FPG, HbA1c, TG, TC, UA, HCY, CRP, albumin, eGFR, baseline NIHSS score, and KWDT. Thrombolysis and GCS were not included.*

*Abbreviations: VIF, variance inflation factor; SAP, stroke-associated pneumonia; NPAR, neutrophil percentage-to-albumin ratio; COPD, chronic obstructive pulmonary disease; FPG, fasting plasma glucose; HbA1c, glycated hemoglobin; TG, triglycerides; TC, total cholesterol; UA, uric acid; HCY, homocysteine; CRP, C-reactive protein; eGFR, estimated glomerular filtration rate; NIHSS, National Institutes of Health Stroke Scale; KWDT, Kubota Water Drinking Test; GCS, Glasgow Coma Scale.*
