# Supplementary material for: Elevated Neutrophil Percentage‐to‐Albumin Ratio Is Associated With Stroke‐Associated Pneumonia in Acute Ischemic Stroke: A Retrospective Cohort Study
Source: Brain Behav. 2026 Nov 5;16(11):e71891. doi: 10.1002/brb3.71891 (PMC13740347; doi:10.1002/brb3.71891)
Supplement: Supplementary file 2 [file BRB3-16-e71891-s002.docx]

**Supplementary Table S2. Model robustness, internal validation of Model 3, and head-to-head biomarker comparison after multiple imputation**

| **Analysis** | **Result** | **P-value** |
| --- | --- | --- |
| ***Part A: Sensitivity analysis - effect of albumin adjustment*** |  |  |
| Linear log2-NPAR (with albumin, 18 covariates) | OR = 6.14 (3.19-11.82) | **<0.001** |
| Linear log2-NPAR (without albumin, 17 covariates) | OR = 6.97 (3.94-12.33) | **<0.001** |
| Piecewise (with albumin): NPAR < 1.758 | OR = 0.61 (0.26-1.43) | 0.259 |
| Piecewise (with albumin): NPAR >= 1.758 | OR = 97.42 (32.07-295.91) | **<0.001** |
| Piecewise (without albumin): NPAR < 1.758 | OR = 0.59 (0.25-1.37) | 0.218 |
| Piecewise (without albumin): NPAR >= 1.758 | OR = 77.68 (28.81-209.49) | **<0.001** |
| Part B: Internal validation of the fully adjusted Model 3 only |  |  |
| Apparent AUC (MI-averaged Model 3) | 0.884 | - |
| Optimism-corrected AUC | 0.875 (95% CI: 0.860-0.887) | - |
| Average optimism | 0.0091 | - |
| AUC excluding albumin (17 covariates) | 0.884 | - |
| ***Part C: Head-to-head biomarker comparison (univariate, full cohort)*** |  |  |
| NPAR | AUC = 0.785 (0.756-0.814); Sens 68.6%, Spec 77.2% | Reference |
| SII | AUC = 0.746 (0.715-0.776); Sens 62.0%, Spec 78.8% | vs NPAR: 0.001 |
| CRP | AUC = 0.826 (0.800-0.851); Sens 66.8%, Spec 86.4% | vs NPAR: 0.019 |
| ***Part D: Model calibration*** |  |  |
| Hosmer-Lemeshow goodness-of-fit (10 groups) | chi-square = 9.37 | 0.312 |

*Analyses were performed in the full imputed analytic cohort (N = 1,766, SAP = 376). HbA1c missing values were handled using multiple imputation (m = 20).*

*Part A: linear and piecewise logistic regression models were fitted in each imputed dataset and pooled using Rubin's rules. The common piecewise threshold was selected from the first completed dataset and then applied across all 20 datasets.*

*Part B: apparent AUC was calculated from the average predicted probability across 20 imputed Model 3 fits. Bootstrap optimism correction was confined to the fully adjusted Model 3 and used 200 resamples in the first completed dataset. The combination models in Table 4 and Figure 3 were not internally validated.*

*Part C: NPAR, SII, and CRP are fully observed in the analytic cohort; AUC 95% CIs and pairwise comparisons were calculated using the DeLong method.*

*Part D: calibration was assessed using the Hosmer-Lemeshow goodness-of-fit test with 10 groups based on MI-averaged predicted probabilities. P > 0.05 indicates adequate calibration.*

*Abbreviations: NPAR, neutrophil percentage-to-albumin ratio; SAP, stroke-associated pneumonia; OR, odds ratio; CI, confidence interval; AUC, area under the ROC curve; SII, systemic immune-inflammation index; CRP, C-reactive protein; PLT, platelet count; NTC, neutrophil count; LTC, lymphocyte count.*
